# Announcement: Eighteenth Conference on Coordination and Bio-Inorganic Chemistry

**DOI:** 10.1155/MBD.2000.291

**Published:** 2000

**Authors:** Milan Melník

**Affiliations:** Department of Inorganic Chemistry Slovak Technical University Radlinského 9 Bratislava 812 37 Slovakia


### EIGHTEENTH CONFERENCE ON COORDINATION AND BIO-INORGANIC CHEMISTRY

CHALLENGES FOR COORDINATION CHEMISTRY IN THE NEW CENTURY
Smolenice , Slovakia
4 – 8 June 2000

The conferences on coordination chemistry held regularly in Slovakia, have a long tradition. The First Conference of this kind was organized in 1964. The Conferences took place in Smolenice Castle that was adapted for the needs of the conferences and similar events and serves now as a Congress Center of the Slovak Academy of Sciences. Many top coordination chemists from all over the world attended the Conferences till now and Smolenice Castle became the place where many new scientific and personal contacts were established.

The Organizing Committee invites you to participate in the 18th International Conference on Coordination and Bio-inorganic Chemistry. The Conference will be organized by the Slovak Chemical Society, Slovak Technical University, Comenius University, and Slovak Academy of Sciences in Bratislava and held in Smolenice Castle between 4 and 8 June, 2001.

Since the Castle is about 60 km far from Bratislava, bus transport from Bratislava to Smolenice and return will be organized on arrival and departure days, respectively. Bratislava can be reached by plane, train, bus (from Vienna or Prague) or ship (from Vienna). Since there is a regular bus connection between Vienna airport and Bratislava bus station (45 km) it is possible to take a plane to Vienna as well.

The Castle is situated in the middle of forests and parks on the southern slopes of the Low Carpathians. It is a wonderful place with a quiet atmosphere that creates ideal preconditions for fruitful and useful discussions. As a rule, the weather in the region is very pleasant in the first decade of the June, with temperatures about 20-25 °C. More information you can find in the website **www.chtf.stuba.sk/kach/smolenice**

The official language of the Conference will be English.

### SCOPE OF THE CONFERENCE

**A** **Structure and reactivity of coordination and bioinorganic compounds**
(Synthesis, structure, stability, kinetics, photochemistry, catalysis)

**B** **Complexes in biological systems, human medicine, and environment**
(Natural a model bio-complexes, relationship between their structures and biological activities, function of therapeutic agents and enzymes, catalyzed destruction of pollutants)

**C** **Applied inorganic chemistry**
(Supramolecular devices, magnetic materials, superconductors, magnetic, electrical and optical properties of inorganic and coordination compounds, application of inorganic catalysts)

**D** **Supramolecular coordination chemistry**
(Solid state systems, host - guest chemistry, self-assembling complexes, dendrimers, inorganic super structures, molecular modeling)

**SCIENTIFIC PROGRAM**

The scientific program is supposed to be divided into four working days. Each of the topics will be introduced by a plenary lecture delivered by an invited lecturer.

The other participants may present their contributions to the scientific program of the Conference as:

- plenary lectures lasting 30 minutes, discussion including,
- section lectures lasting 15 minutes, discussion including.

Young scientists are offered to use the possibility to present their results as posters or section lectures lasting 10 minutes. However, each participant can deliver only one lecture/poster.

**CONTRIBUTED PAPERS**

Each participant is expected to submit one paper only. One-page abstracts of all contributions will be published in the Proceedings of the 18th International Conference on Coordination and Bio-inorganic Chemistry. Those participants who wish to present 30 minute lectures, are required to submit their contributions as a six pages paper that will be published in a monograph entitled "Challenges for Coordination Chemistry in the New Century" and printed by the Slovak Technical University Press in Bratislava. The papers will be reviewed by referees and their recommendations will be taken into consideration by the editors. The contributions of those who intend to present posters, will be published in the Proceedings only.

The participants will receive the Proceedings as well as the monograph together with reprints of their papers before the Conference at the registration.

**SOCIAL PROGRAM**

Social program, including a welcome party and final banquet, is planned. Besides that, a half-day optional excursion (cultural and social event) to Bratislava will be organized.

**ACCOMMODATION**

Accommodation for all participants will be arranged in Smolenice Castle. Since there are only 90 beds available for participants in the Castle the number of participants is obviously limited by this number. Consequently, those applicants will be preferred who show their unambiguous interest to participate and send all required materials in due time.

(Further information in the Second Circular.)

**FEES**

1) The registration fee is 150 USD.

This sum will cover all expenses connected with monograph, proceedings of the conference, reprints, conference materials, services.

2) The Conference fee is 150 USD.

It includes all expenses for board in Smolenice Castle (three complete meals per day, coffee & tea), social program, services during the Conference, travel expenses from Bratislava to Smolenice and return.

3) The lodging in Smolenice Castle will be paid separately.

Thanks to the sponsorship of the Slovak Academy of Sciences, the sum paid for five nights should not exceed 200 USD (depending on the category of rooms).

**PRELIMINARY APPLICATION**

You can apply via internet or a Preliminary Application Form can be obtained from

**Professor Milan MELNÍK**
**Department of Inorganic Chemistry, Slovak Technical University**
**Radlinského 9,**
**812 37 BRATISLAVA, Slovakia**
**Telephone: +421 7 5249 5257**
**Fax: +421 7 5249 3198**
**<sirota@cvt.stuba.sk>**

Your application must reach the Organizing Committee **before December 20, 2000.**

